# Is a Cholera Outbreak Preventable in Post-earthquake Nepal?

**DOI:** 10.1371/journal.pntd.0003961

**Published:** 2015-08-13

**Authors:** Eric J. Nelson, Jason R. Andrews, Stacey Maples, Michele Barry, John D. Clemens

**Affiliations:** 1 Department of Pediatrics, Stanford University School of Medicine, Stanford, California, United States of America; 2 Department of Medicine, Stanford University School of Medicine, Stanford, California, United States of America; 3 Stanford Geospatial Center, Stanford University Libraries, Stanford, California, United States of America; 4 International Centre for Diarrhoeal Disease Research, Bangladesh, Dhaka, Bangladesh; University of Queensland, AUSTRALIA

**Fig 1:**
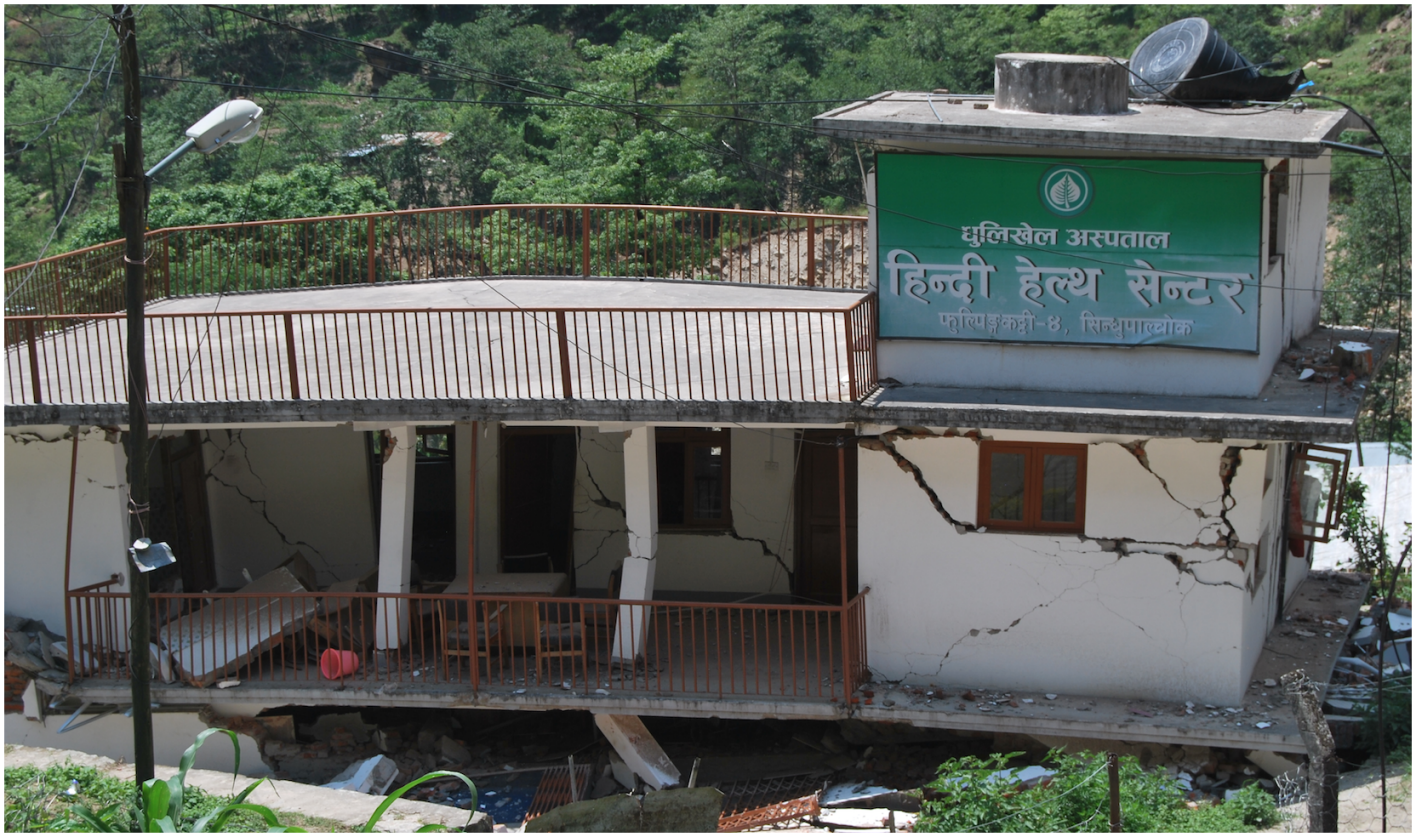
Collapsed outreach center of Dhulikhel Hospital, Kathmandu University Hospital (http://www.dhulikhelhospital.org/). This image highlights a water tank that has fallen off the pedestal on the roof of a clinic. The inability to safely store water is one of many reasons why clean water has become scarce across Nepal. Photograph courtesy of U.u.H. Schmel and R.K. Mahato.

The 2010 earthquake in Haiti and the subsequent cholera outbreak taught us multiple lessons on how we might better avert cholera outbreaks, beyond simply improving access to clean water and sanitation [1]. Post-earthquake Nepal is now a test of how well we learned these lessons (Fig 1). Earthquake-associated mortality generally progresses in well-defined phases that ultimately give way to longer-lasting periods of new and complex health needs. The first phase is caused by the immediate kinetic trauma. The second is a product of infectious complications from the bodily trauma. The third is linked to infectious disease outbreaks enabled by the destroyed infrastructure; many of these latter diseases are now vaccine preventable, including cholera.

The most recent oral cholera vaccine (Shanchol) is designed to be a low-cost yet high-quality vaccine that can be deployed for mass immunization in settings at risk of outbreaks [2,3]. Efficacy is estimated to be 65% for at least five years [4,5], and herd immunity (protection of non-immunized people) may push efficacy higher at the population level [6]. Pre-emptive mass vaccination for cholera after the Haitian earthquake did not occur because of two factors [1]. The first was a logical rationale that cholera had not been in Haiti and therefore was not anticipated. The second factor was that the available vaccine supply was not sufficient for mass vaccination. Sadly, an epidemic of cholera did occur in Haiti with devastating sequelae.

After the Haitian cholera outbreak, the World Health Organization (WHO) and partners led an ambitious effort to build an oral cholera vaccine stockpile that could be used to vaccinate communities impacted by emergencies to potentially avert what happened in Haiti. Such a stockpile of the newer vaccine Shanchol does exist now [2]. The Shanchol vaccine is relatively low cost ($US1.85 per dose) compared to the older vaccine Dukoral ($US4.7–$US9.4 per dose), and thoughtful administration could prevent the spread of cholera. However, the available number of doses still remains relatively limited [7].

**Fig 2:**
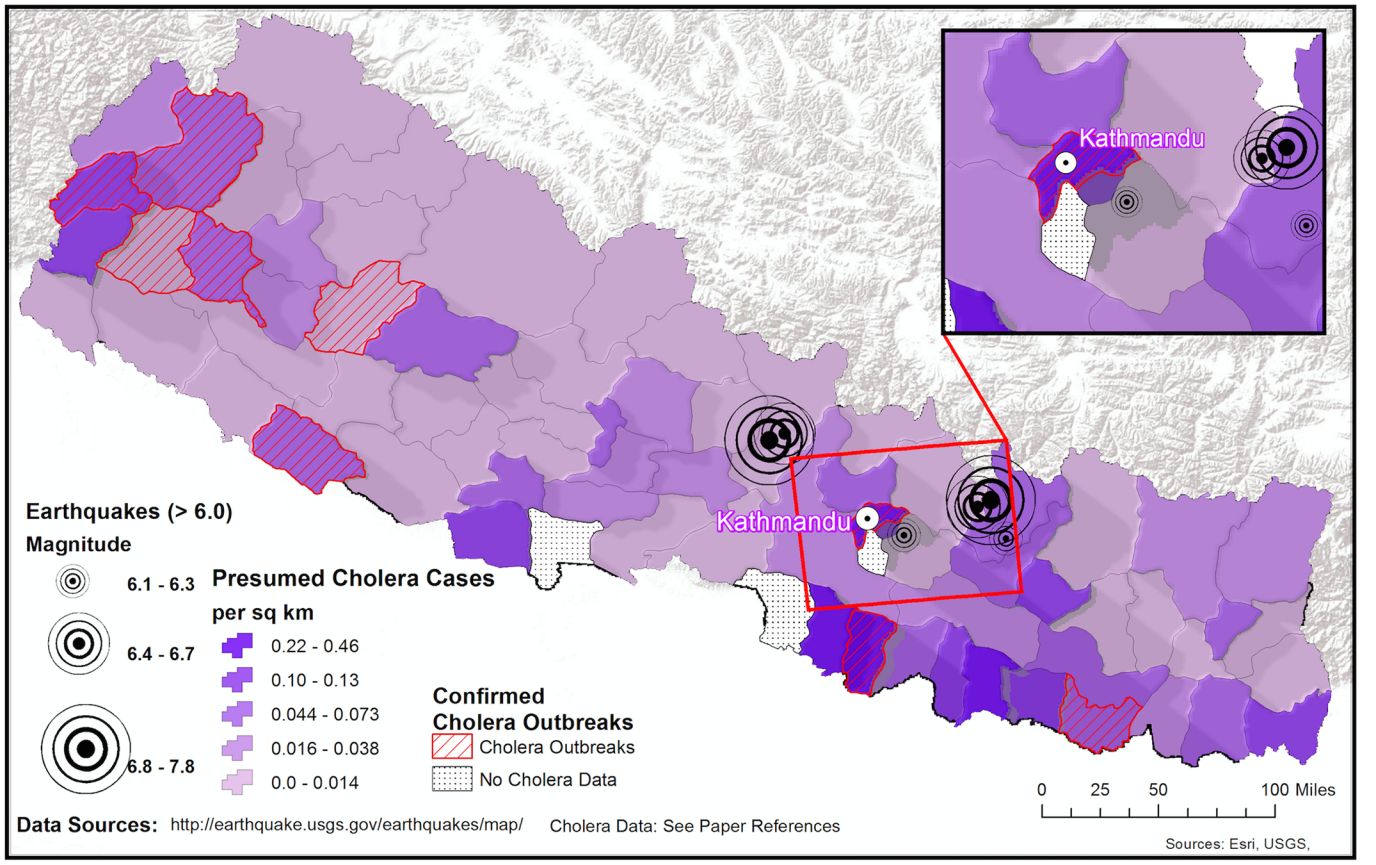
Regions with historical cholera outbreaks in Nepal. Distribution of confirmed cholera outbreaks (hashed areas) [8–16]. Also shown are presumed cholera cases for 2010–2014 based on syndromic clinical observation [19]; the median and mean incidence were 10.4 and 19.1 cases per 100,000 population, respectively (range = 0–107; first quartile was 4.6 and third quartile 24.3). The incidence in Kathmandu was 15.1.

**Fig 3:**
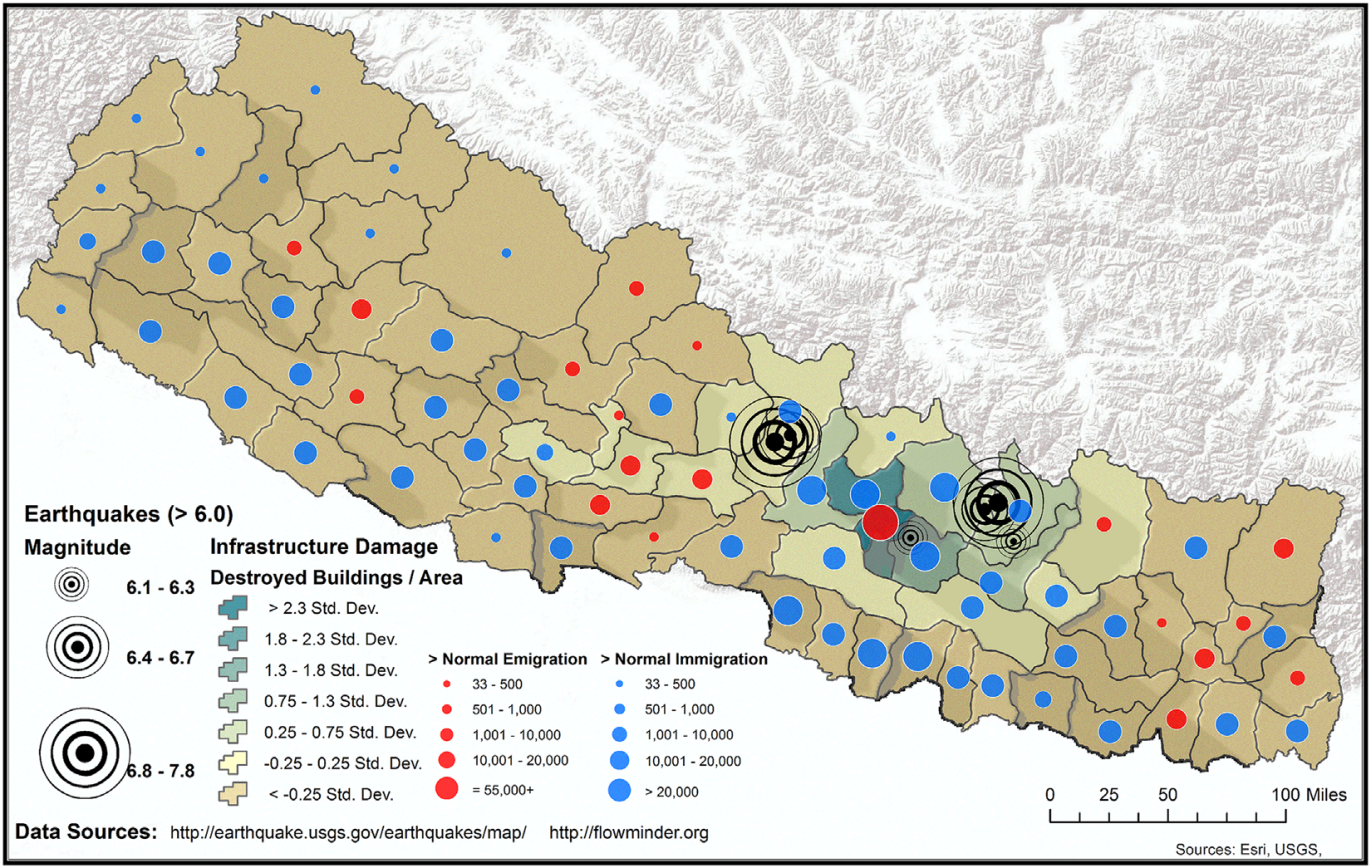
Internally displaced persons and infrastructure damage in post-earthquake Nepal. Earthquake-affected regions in Nepal and post-earthquake human migratory patterns; publically available data sources are referenced in the figure.

There are at least two viewpoints on how a cholera vaccine should be implemented in post-earthquake Nepal. One opinion is that a “pre-emptive” cholera vaccination campaign in Nepal is indicated given that cholera is endemic [8–16], water and sanitation infrastructure are collapsed, medical systems lack the capacity to mount a second major response, and the monsoon season will raise the risk of an outbreak. That said, a “wait-and-see” approach may also be reasonable. For example, most cholera outbreaks in Nepal historically have occurred away from the areas most heavily affected by the recent earthquakes [8–16], and the actual risk of a cholera outbreak may still be low to moderate. However, Kathmandu has frequently reported cases of cholera, and *Vibrio cholerae* are present in surface waters in the capital [8]. Moreover, the post-earthquake migration out of the Kathmandu valley to rural villages and the anticipated return of populations from these areas will likely blur the historical lines of locally endemic and non-endemic regions for cholera (Figs 2 and 3). For these reasons, predicting if and where a cholera outbreak may occur in Nepal is very difficult.

We acknowledge the conflict between a “pre-emptive” versus “wait-and-see” decision. We recommend that health officials and non-government organizations at least immediately deploy a low-cost early warning system for cholera and other actionable waterborne diseases (e.g., shigellosis and typhoid). In the “wait-and-see” situation, an early warning system will provide a mechanism to actively fulfill the WHO mass vaccination criteria [2]. In the “pre-emptive” scenario, an early warning system is more of a monitoring and evaluation mechanism to study the impact of the vaccination campaign.

Deploying an early warning system in post-earthquake Nepal poses its own challenges. There are limited systems and laboratory capacity for active disease surveillance, particularly in the most affected areas outside the Kathmandu Valley. In addition, there is nuance to when and where cholera outbreaks strike and variability in the type of strains and drug resistance patterns that drive these outbreaks (Fig 2) [8–16]. Moreover, the hyper-locality of outbreaks can make facility-based surveillance insufficient when it does not reach all areas; this is particularly problematic when a large portion of the population is receiving care through medical relief camps, which are often not integrated into formal reporting systems.

Fortunately, teams are asking how both low- and high-technology systems might be leveraged to meet these urgent needs. Chief among these needs is navigating the challenge of identifying the highest-risk populations amid rapidly changing landscapes, for which traditional paper-based reporting systems may be too slow. Cellular technology solutions are clearly in vogue in this era of global health, but they do offer several key opportunities during disasters. The most important advantage is that cell towers are built strong and elevated well off the ground, have an independent generator, and are secure. These cell towers have shown amazing resilience through floods, tsunamis, and earthquakes. In fact, they are frequently one of the few reliable resources that remain after disasters. In Nepal, cellular networks were largely functional immediately following the earthquake, while grid electricity and water supply were interrupted for days in most affected areas.

There are several models for how an early warning system for cholera might be designed and deployed in post-earthquake Nepal: (i) One high-tech model is to place smartphone-dependent surveillance tools at triage in healthcare facilities. These devices can provide both decision support and real-time reporting; however, smartphones have higher electrical demands than feature phones (e.g., Nokia flip phone) and the 3G networks are limited to areas with high population density in the valleys. (ii) A medium-tech model is a hotline model. In this model, a paper-based reporting system is deployed at triage. Cases that meet criteria for a suspected cholera case can be reported to a hotline that, in turn, triages the case, enters data electronically, and coordinates the appropriate clinical and epidemiologic response. Alternatively, the reporting can be done by sending a text message. This medium-tech system is more durable because it relies only on feature phones. (iii). One low-tech option is a traditional paper-based system that compiles paper-based reports at triage and sends the reports to a central surveillance team on a regular basis by ground transport. Although slow in mountainous areas, the system may be the only option in settings where basic cellular signal does not penetrate. Each model has its strengths and weakness, and it is the purview of the local surveillance team to design a strategy that will work best in its catchment yet still efficiently share findings with the WHO and government authorities.

The efficacy of any surveillance system is only as great as the quality of the data collected. Acute watery diarrhea is non-specific, particularly among children under two years of age, and systems aggregating and reporting all acute diarrheal cases are likely to be highly noisy. The WHO case definitions for suspected cholera in non-epidemic settings are more specific and may help focus limited laboratory resources [17]. Cholera rapid diagnostic tests can be performed in settings with minimal laboratory capacity, and their sensitivity and specificity can be enhanced with an enrichment step [18], but culture should still be performed to confirm an outbreak. Efficient systems for transporting specimens to central laboratories for culture and identification are needed, which is a non-trivial challenge given Nepal’s topography and transportation infrastructure. Finally, all data, independent of the surveillance mechanism, should be aggregated, analyzed, and visualized in a manner that enables stakeholders to make rapid, evidence-based decisions. This may be the most important and difficult component of the surveillance system, and yet it is often the part that is most neglected. Technical support is needed to prepare data analysis plans and platforms, determine geographical and temporal windows for analysis, set thresholds for defining outbreaks, and ensure that all analyses are continuously performed, interpreted, and conveyed to the relevant public health decision-makers.

In conclusion, it is the willingness to deploy a spectrum of strategies that are both locally fieldable yet coordinated that will provide life-saving surveillance in the gap when traditional systems are disrupted. In addition, the development and deployment of robust surveillance systems may also fill a critical need even when the infrastructure recovers by reducing time lags in reporting and data interpretation.

It may not be possible to prevent a small outbreak in Nepal; however, we advocate that a diverse and nimble toolkit (e.g., education, hygiene, water, and sanitation campaigns, coordinated early warning systems) may help prevent a large-scale cholera outbreak. We also advocate for continued funding of the global vaccine stockpile to ensure that the threshold to use the stockpile is a function of the needs of the people and not of the cost and logistics of deciding who gets the vaccine and who does not.

In the coming weeks, stakeholders will be deciding whether to pursue a cholera vaccination campaign for Nepal. The more data they have from early warning systems, the less difficult their decision will be.
